# Advances in Fatigue Analysis and Numerical Simulation in Engineering Materials

**DOI:** 10.3390/ma19132705

**Published:** 2026-06-23

**Authors:** Mustafa Awd, Frank Walther

**Affiliations:** 1 Process Informatics Group, Department of Mechanical & Materials Engineering, University of Turku, ICT-City, Joukahaisenkatu 3-5, FI-20520 Turku, Finland; 2 Chair of Materials Test Engineering (WPT), TU Dortmund University, Baroper Straße 303, D-44227 Dortmund, Germany; frank.walther@tu-dortmund.de

The field of materials science and engineering is undergoing a transformative era, driven by the convergence of advanced manufacturing, experimental characterization, and computational modeling. The articles in this Special Issue illustrate the breadth and depth of current research, which ranges from microstructural analyses and the development of new methods to predictive models for understanding surface and bulk integrity in metals and alloys. This Editorial summarizes the key findings from these studies and, drawing on additional recent contributions, places them within the broader context of ongoing advancements.

Accurately predicting fatigue life remains a key challenge in the field of materials science and engineering. Mrozinski et al. [1] present a robust framework for predicting fatigue crack initiation and growth, leveraging both experimental data and computational models. Their approach underscores the importance of integrating microstructural features and loading histories to improve reliability in life assessments. Stanco et al. [2] extend this paradigm by identifying critical microstructural parameters that govern fatigue performance, highlighting the interplay between grain size, inclusion content, and local stress fields.

Domanski et al. [3] introduce Voronoi-based modeling to simulate microstructural heterogeneity and its impact on crack propagation. This method enables the quantification of variability in the fatigue response, which is crucial for the design of additively manufactured (AM) components. Zichil et al. [4] focus on the role of notches and geometric discontinuities, demonstrating how local stress concentrations can dramatically reduce fatigue life, especially in high-strength alloys.

The integration of artificial intelligence (AI) and machine learning (ML) is rapidly advancing the predictive capabilities of fatigue models. Awd et al. [5] showcase the application of ML algorithms to large experimental datasets, enabling the identification of hidden patterns and the development of more accurate life prediction tools. Their work is complemented by recent studies such as the study by Gbagba et al. [6], who review the state of the art in ML-driven fatigue analysis, and Hills et al. [7], who demonstrate the sensitivity of fatigue strength predictions to input feature selection in AM Ti-6Al-4V. Related mechanistic ML approaches further show how first-principles models and process metadata can be combined for fatigue strength design in additive manufacturing [8]. Additional recent contributions also reflect the growing role of data-driven and structurally informed approaches in AM materials research [9,10].

Residual stress is a pervasive phenomenon in both conventionally manufactured and AM materials, influencing crack initiation, propagation, and overall structural integrity. Abyazi et al. [11] investigate the effect of spherical inclusions on residual stress distribution and fatigue behavior, providing insights into the mechanisms by which microstructural inhomogeneities can localize damage. Alshoaibi et al. [12] employ numerical analysis to elucidate the interplay between residual stress fields and fatigue crack growth, offering guidance for process optimization and post-processing treatments.

The importance of advanced experimental techniques is further highlighted by Ao et al. [13], who utilize four-dimensional (4D) characterization methods to capture the evolution of microstructure and residual stress during cyclic loading. Their findings reveal the dynamic nature of damage accumulation and underscore the need for in situ monitoring in fatigue-critical applications. Atzori et al. [14] revisit classical fracture mechanics approaches, emphasizing the continued relevance of notch sensitivity and stress intensity factors in modern materials design.

The intricate relationship between microstructure and mechanical behavior is a recurring theme across these studies. Recent work by Liu et al. [15] and Munther et al. [16] explores the use of surface treatments such as laser shock peening to induce beneficial compressive residual stresses and grain refinement, thereby enhancing fatigue life. Similarly, Grima et al. [17] and Maleki et al. [18] investigate the effects of shot peening and severe plastic deformation, respectively, on the microstructure and mechanical response of AM and wrought alloys.

Additive manufacturing continues to revolutionize the production of complex components, but also introduces new challenges related to process-induced defects, anisotropy, and residual stress. Kudrna et al. [19] compare residual stress distributions in parts produced by selective laser melting (SLM) and wire arc additive manufacturing (WAAM), revealing the influence of thermal history and post-processing on structural integrity. Louw et al. [20] provide a framework for quantifying the degradation of fracture toughness due to process-induced residual stresses in laser powder bed fusion (LPBF) alloys. Complementary studies on PBF-LB AlSi10Mg further demonstrate that anomaly data can be transferred to fatigue property prediction across parts with different volumes, highlighting the importance of defect-informed assessment strategies [21].

The mitigation of process-induced defects is addressed by Moser et al. [22], who evaluate post-processing methods such as blasting and vibratory finishing for removing interruption marks and improving fatigue strength in AM components. Manchili et al. [23] examine the trade-offs between surface quality and residual stress in electrochemically post-processed 316L stainless steel, highlighting the need for integrated process optimization. Related work on powder-mixture-based AM tool steels likewise shows that post-processing strongly influences microstructure evolution and mechanical performance [24].

The articles in this Special Issue collectively illustrate the multifaceted nature of fatigue, residual stress, and microstructure–property relationships in modern materials. The integration of advanced modeling, experimental, and data-driven approaches is enabling unprecedented insights, yet significant challenges remain. The stochastic nature of microstructural features, the complexity of multi-axial loading, and the influence of environmental factors all demand continued innovation in both methodology and application.

Emerging directions include the use of 4D and in situ characterization techniques [13], the development of multiscale and probabilistic models [25,26], and the application of AI/ML for real-time process monitoring and life prediction [5,6]. The ongoing refinement of surface treatments and post-processing methods [16,17,18] will be essential for maximizing the performance and reliability of next-generation materials and structures.

In conclusion, the research presented here not only advances our understanding of the fundamental mechanisms governing fatigue and residual stress but also provides practical guidance for the design, manufacturing, and maintenance of critical components. As the field continues to evolve, the synergy between experimental, computational, and data-driven approaches will be key to unlocking new levels of performance and safety in engineering materials.
